# Evaluating the triglyceride glucose index as a predictive biomarker for osteoporosis in patients with type 2 diabetes

**DOI:** 10.3389/fendo.2025.1534232

**Published:** 2025-04-07

**Authors:** Jinxiang Zhan, Qipeng Wei, Weijun Guo, Zihao Liu, Shiji Chen, Qingyan Huang, Shuang Liang, Dongling Cai

**Affiliations:** ^1^ Department of Orthopedics, Panyu Hospital of Chinese Medicine, Guangzhou, Guangdong, China; ^2^ Panyu Hospital of Chinese Medicine, Guangzhou University of Chinese Medicine, Guangzhou, Guangdong, China

**Keywords:** triglyceride glucose index, type 2 diabetes mellitus, osteoporosis, risk factor analysis, prediction model

## Abstract

**Objective:**

Osteoporosis is a common condition among individuals with type 2 diabetes; however, the relationship between insulin resistance, as measured by the Triglyceride Glucose Index (TyG), and osteoporosis has not been sufficiently explored. This study seeks to address this research gap by investigating the diagnostic value of TyG in identifying osteoporosis in patients with type 2 diabetes.

**Methods:**

A retrospective analysis was performed on clinical data from 207 diabetic subjects (83 in the osteoporosis group, 124 in the non-osteoporosis group), using SPSS version 27.0 and MedCalc 23 for statistical analysis.

**Results:**

Significant statistical differences were noted between the two groups in terms of gender, age, hemoglobin levels, red blood cell count, total cholesterol levels, and the TyG. Binary logistic regression analysis revealed that gender, age, and TyG are independent predictors of osteoporosis in patients with type 2 diabetes. Receiver operating characteristic (ROC) analysis showed that the area under the curve for TyG, gender, age, and their combination in predicting osteoporosis among patients with T2DM was 0.653, 0.698, 0.760, and 0.857, respectively. Additionally, the diagnostic performance of the TyG value was effectively evaluated, determining 8.78 as the optimal cutoff value, with a corresponding sensitivity of 89.1% and specificity of 52.4%. Meanwhile, the predictive model constructed using gender, age, and the TyG index achieved an area under the curve (AUC) of 0.857 (95% confidence interval: 0.801~0.901), with a maximum Youden index of 0.629. The corresponding diagnostic sensitivity was 83.1% and the specificity was 79.8%.

**Conclusion:**

The TyG holds potential to serve as a prominent biomarker for the diagnosis of osteoporosis among type 2 diabetic patients in various clinical settings.

## 1 Introduction

Osteoporosis is a systemic skeletal disorder characterized by a reduction in bone mass, deterioration of bone microarchitecture, increased fragility, and heightened susceptibility to fractures (1, 2). At the same time, studies have shown that impaired bone microarchitecture and reduced bone turnover are important causes of increased fracture risk (3). It has emerged as a significant public health concern, particularly among individuals aged 50 and older in China (4). The insidious onset of osteoporosis complicates early diagnosis, and the subsequent development of osteoporotic fractures adversely impacts the quality of life of affected individuals (5). Type 2 Diabetes Mellitus (T2DM) is a chronic metabolic disease characterised by insulin resistance and defective insulin secretion, mainly manifested by elevated blood glucose. Both T2DM and osteoporosis are prevalent chronic diseases with distinct pathogenic mechanisms. T2DM is primarily attributed to insulin resistance and a relative deficiency in insulin secretion, influenced by a combination of genetic and environmental factors, as well as chronic inflammation (6, 7). In contrast, osteoporosis results from an imbalance in the bone remodeling process, alterations in hormonal levels, and nutritional deficiencies, all of which contribute to decreased bone density (8). Insulin plays a critical role in bone metabolism; consequently, insulin resistance may lead to diminished bone formation and increased bone resorption, thereby heightening the risk of osteoporosis (9). Research indicates that insulin resistance may negatively affect bone density by impairing the function of both osteoblasts and osteoclasts (10).Furthermore, insulin resistance may influence bone metabolism through the action of pro-inflammatory cytokines and oxidative stress, potentially initiating the progression of osteoporosis (11–13). Both conditions have substantial implications for contemporary healthcare, significantly increasing medical costs. Therefore, there is an urgent need to develop comprehensive management strategies and further research on preventive measures and innovative treatments for osteoporosis in patients with T2DM in order to improve their quality of life.

The prevalence of T2DM patients who also exhibit osteoporosis is increasing in clinical settings, accompanied by a rising incidence of undiagnosed early-stage osteoporosis. Current screening methods for osteoporosis remain underutilized, resulting in missed opportunities for timely intervention. Consequently, the implementation of early osteoporosis screening in individuals with T2DM is of paramount importance. In 2008, Guerrero-Romero and colleagues introduced the Triglyceride Glucose Index (TyG) as a metric for identifying insulin resistance (IR) (14). Subsequent clinical studies have substantiated the reliability of TyG as a measure for assessing insulin resistance in at-risk populations, particularly in the context of predicting high-risk groups for diabetes (15–18).

This study aims to address a significant gap in the literature by investigating TyG as a potential biomarker for osteoporosis in patients with T2DM. Previous studies have explored the individual effects of insulin resistance and diabetes on bone health, but few studies have specifically investigated the diagnostic value of TyG in this regard. By focusing on TyG, our study provides a novel perspective that integrates metabolic health with bone density assessment, thus potentially enhancing screening strategies.

In addition, this study contributes to the existing knowledge by providing empirical evidence of an association between elevated TyG levels and decreased bone mineral density in diabetic patients. The findings suggest that TyG can be used as a simple, cost-effective tool for the early identification of osteoporosis, which is essential for timely intervention and management. This research not only enhances our understanding of the metabolic factors influencing bone health but also proposes a practical approach for early osteoporosis screening in individuals with T2DM.

## 2 Information and methods

### 2.1 Study population and diagnostic criteria for osteoporosis

This study retrospectively analyzed the clinical records of 207 patients diagnosed with T2DM who were admitted to the Spinal Surgery Department of Panyu District Traditional Chinese Medicine Hospital between January 2021 and August 2024. Due to the retrospective nature of the study, the Ethics Committee of Panyu Traditional Chinese Medicine Hospital waived the need of obtaining informed consent. Meanwhile, all methods were carried out in strict accordance with relevant guidelines and regulations, and all experimental protocols were duly approved by the Ethics Committee of Panyu Traditional Chinese Medicine Hospital. Bone density of the patients was measured with a Prodigy DXA scanner of General Electric Company of the United States of America, and bone density of lumbar spine, bone density of femoral neck, and bone density of the left hip joint were recorded. Referring to the World Health Organization (WHO) diagnostic criteria for osteoporosis, a T score of ≤-2.5 at any site was considered to be diagnostic of osteoporosis.

### 2.2 Inclusion and exclusion criteria

Inclusion criteria were as follows:

Diagnosis of T2DM.
Age of 50 years or older.
Availability of complete clinical data, including the patient’s age, laboratory test results, and other relevant information.

Exclusion criteria were as follows:

History of fractures.
Previous spinal surgeries (e.g., vertebroplasty or lumbar fusion).
Long-term use of medications affecting glucose and lipid metabolism (e.g., glucocorticoids).
Other endocrine disorders impacting bone metabolism (e.g., thyroid, parathyroid, or gonadal disorders).
Diagnosis of infectious diseases or blood disorders.
History of cancer, including multiple myeloma and thyroid cancer.
Severe liver or kidney dysfunction.
Diagnosis of type 1 diabetes.
Patients taking osteoporosis medication.
Patients receiving lipid-lowering medication treatment.

### 2.3 Data collection and analysis

Dual-energy X-ray bone mineral density and biochemical index results for all 207 subjects were retrospectively reviewed. The objective of this study was to collect and record data on gender and age of patients in both groups. Patients were required to fast for a minimum of 8 hours overnight prior to venous blood sample collection in the morning. The following tests were conducted: blood routine tests [including red blood cells (RBC), hemoglobin (Hb), platelets (PLT), white blood cells (WBC), neutrophils (NEUT), lymphocytes (LYM), and monocytes (MONO)]; creatinine (Cr); lipid profile tests [including triglycerides (TG), total cholesterol (TC), high-density lipoprotein cholesterol (HDL-C), and low-density lipoprotein cholesterol (LDL-C)]; albumin (ALB); and coagulation tests [including quantitative fibrinogen (FIB) and quantitative D-dimer (D-D)]. Additionally, TyG was calculated using the following formula: TyG = ln[(TG (mg/dL) × FPG (mg/dL))/2].

### 2.4 Statistical analyses

Data analysis was performed using SPSS version 27.0 statistical software, with a significance level set at α = 0.05, where p < 0.05 was considered statistically significant. For continuous variables with a normal distribution, independent t-tests or analysis of variance (ANOVA) were used; for continuous variables with a non-normal distribution, Mann-Whitney U test were employed. Categorical variables were analyzed using chi-square tests or Fisher’s exact tests. Prior to conducting binary logistic regression analysis, multicollinearity among independent variables was assessed by calculating the variance inflation factor (VIF) and tolerance values. VIF values were well below 10, and all tolerance values exceeded 0.1, indicating no significant multicollinearity. Following this, binary logistic regression analysis was performed with the presence of osteoporosis in patients with T2DM as the dependent variable. Independent variables included those that were statistically significant in the univariate analysis. Additionally, receiver operating characteristic (ROC) curve analysis was conducted using MedCalc 23, with the maximum Youden index calculated using the formula: Youden index = Sensitivity + Specificity - 1 (19).

## 3 Results

### 3.1 Baseline characteristics

A total of 207 patients diagnosed with T2DM were enrolled in the study, comprising 83 individuals in the osteoporosis group and 124 individuals in the non-osteoporosis group. Comparative analysis of general characteristics revealed statistically significant differences between the two groups in terms of gender, age, hemoglobin levels, red blood cell count, total cholesterol levels, and TyG (P < 0.05). However, no significant differences were observed between the groups in white blood cell count, platelet count, neutrophil count, lymphocyte count, monocyte count, creatinine levels, triglyceride levels, high-density lipoprotein cholesterol, low-density lipoprotein cholesterol, albumin levels, quantitative fibrinogen, and D-dimer quantification (see **Table 1**).

**Table 1:** Comparison of clinical data between osteoporosis group and non osteoporosis group.

	Osteoporosis group (n=83)	Non osteoporosis group (n=124)	*P*
Gender			<0.001
Men	10 (12.0%)	53 (42.7%)	
Women	73 (88.0%)	71 (57.3%)	
Age	71.83 ± 8.88	65.28 ± 9.43	<0.001
WBC (10^9^/L)	6.83 ± 2.00	7.17 ± 2.07	0.238
Hb (g/L)	124.77 ± 17.02	130.52 ± 15.46	0.013
RBC (10^12^/L)	4.30 ± 0.62	4.46 ± 0.52	0.048
PLT (10^9^/L)	237.70 ± 64.93	243.46 ± 68.52	0.546
NEUT (10^9^/L)	4.41 ± 1.94	4.32 ± 1.69	0.226
LYM (10^9^/L)	1.85 ± 0.67	2.12 ± 1.46	0.127
MONO (10^9^/L)	0.44 ± 0.14	0.52 ± 0.43	0.097
Cr (μmol/L)	70.87 ± 31.14	71.08 ± 20.64	0.953
TC (mmol/L)	4.87 ± 1.31	4.45 ± 1.19	0.018
TG (mmol/L)	1.82 ± 0.76	1.57 ± 1.67	0.148
HDL-C (mmol/L)	1.23 ± 0.29	1.32 ± 0.64	0.190
LDL-C (mmol/L)	2.86 (2.15,3.65)	2.65 (1.73,3.41)	0.087
ALB (g/L)	40.30 ± 3.39	40.58 ± 4.54	0.631
FIB (g/L)	3.46 ± 1.10	3.59 ± 1.62	0.552
D-D (mg/L)	1.25 ± 1.81	1.16 ± 2.53	0.787
TyG	9.12 (8.92,9.46)	8.77 (8.50,9.05)	<0.001

WBC,white blood cells; Hb,hemoglobin; RBC,red blood cells; PLT,platelets; NEUT,neutrophils; LYM,lymphocytes; MONO,monocytes; Cr,creatinine; TC,total cholesterol; TG,triglycerides; HDL-C,high-density lipoprotein cholesterol; LDL-C,low-density lipoprotein cholesterol; ALB,albumin; FIB,quantitative fibrinogen; D-D,quantitative D-dimer; TyG,Triglyceride Glucose Index.

### 3.2 Predictors for osteoporosis in patients with T2DM

The presence of osteoporosis in patients diagnosed with T2DM was designated as the dependent variable. A binary logistic regression model was employed, incorporating gender, age, hemoglobin levels, red blood cell count, total cholesterol levels, and TyG as independent variables. The results indicate that the odds ratio for males is 0.233 (95% confidence interval: 0.094 - 0.576, p = 0.002), the odds ratio for age is 1.106 (95% confidence interval: 1.059 - 1.156, p < 0.001), and the odds ratio for TyG is 12.282 (95% confidence interval: 4.739 - 31.833, <0.001). The analysis suggests that gender, age, and TyG are independent predictors of osteoporosis in patients with T2DM (see **Table 2**).

**Table 2:** Binary logistic regression analysis of T2DM with osteoporosis.

	B	SE	Wald	OR	95%CI	*p*
Gender	−1.458	0.463	9.922	0.233	0.094 - 0.576	0.002
Age	0.101	0.022	20.342	1.106	1.059 - 1.156	<0.001
Hb	−0.005	0.015	0.096	0.995	0.967 - 1.025	0.757
RBC	−0.286	0.386	0.550	0.751	0.353 - 1.601	0.458
TC	0.119	0.161	0.543	1.126	0.821 - 1.545	0.461
TyG	2.508	0.486	26.643	12.282	4.739 - 31.833	<0.001

Hb,hemoglobin; RBC,red blood cells; TC,total cholesterol; TyG,Triglyceride Glucose Index.

### 3.3 ROC analysis

**Figure 1:**
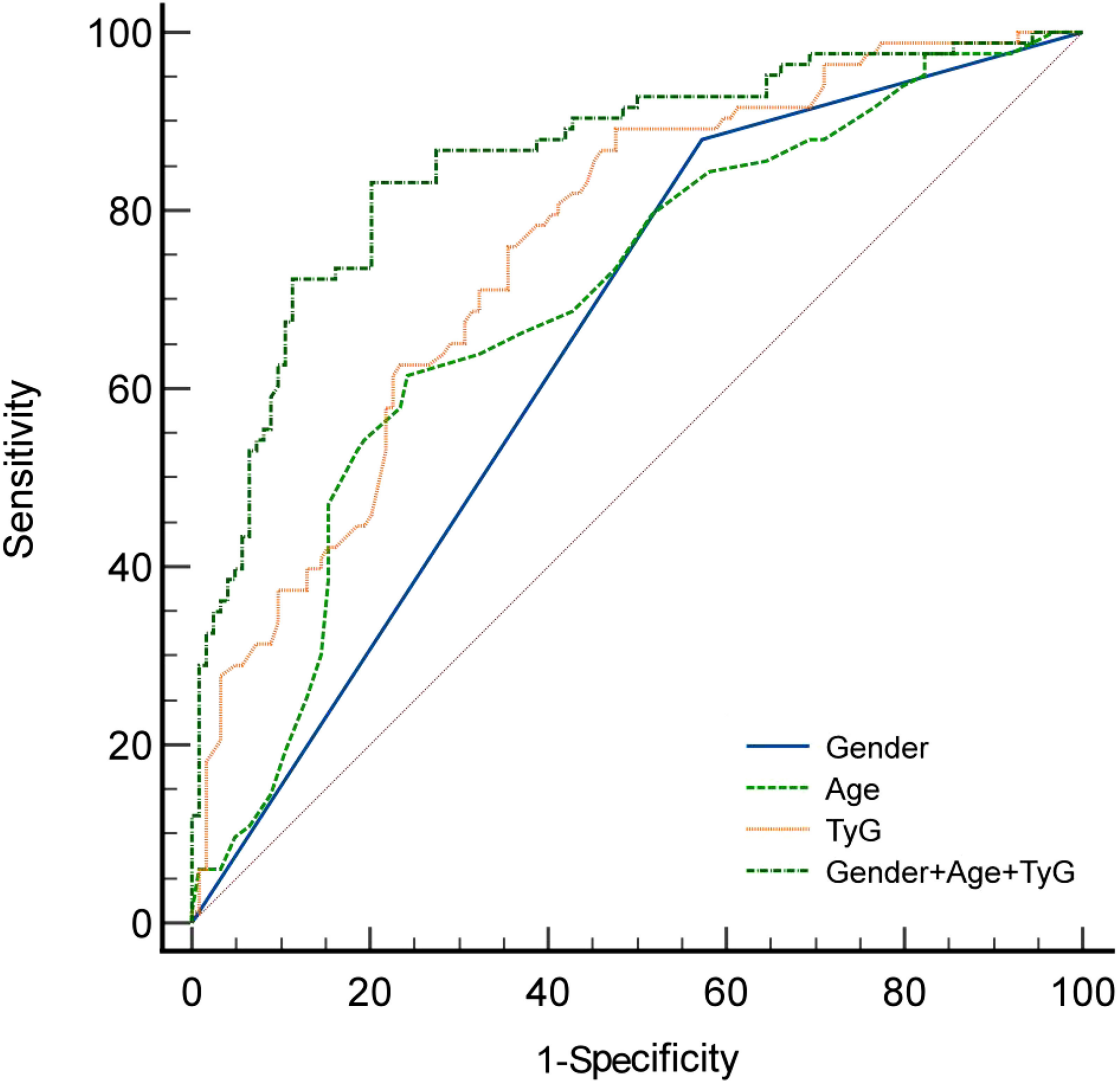
ROC curve of T2DM with osteoporosis.

The receiver operating characteristic (ROC) analysis demonstrated that the area under the ROC curve (AUC) for gender, age, the Triglyceride Glucose Index (TyG), and their combined assessment for osteoporosis in individuals with T2DM were 0.653, 0.698, 0.760, and 0.857, respectively (see **Table 3**, **Figure 1**). Through AUC analysis, the predictive accuracy of the TyG index was evaluated, and the optimal TyG cutoff value was determined using Youden’s Index. The results indicated that a TyG value of 8.78 produced the highest Youden’s Index, signifying an optimal balance between sensitivity and specificity, thus establishing this value as the cutoff. In summary, by generating ROC curves, the diagnostic performance of the TyG value was effectively assessed, identifying 8.78 as the optimal cutoff, with a sensitivity of 89.1% and specificity of 52.4% (see **Table 3**). Meanwhile, the predictive model constructed using gender, age, and the TyG index achieved an area under the curve (AUC) of 0.857 (95% confidence interval: 0.801 - 0.901), with a maximum Youden index of 0.629. The corresponding diagnostic sensitivity was 83.1% and the specificity was 79.8% (see **Table 3**). Meanwhile, the positive predictive value (PPV) and negative predictive value (NPV) of the combined model were also calculated.The PPV was 73.37% and NPV was 87.0%. The positive likelihood ratio (LR+) was 4.11 and the negative likelihood ratio (LR-) was 0.212, indicating that the combined model significantly increased the likelihood of correctly identifying patients with osteoporosis. Additionally, the results of the DeLong test indicated that the combined diagnostic model significantly outperformed the individual use of gender, age, and the triglyceride-glucose index (TyG), with P-values of <0.0001, <0.0001, and 0.0010, respectively.

**Table 3:** Sensitivity analysis of T2DM with osteoporosis.

	AUC	95%CI	Sensitivity	Specificity	Youden index	Optimal threshold
Gender	0.653	0.584~0.718	87.9%	42.7%	0.306	0
Age	0.698	0.631~0.760	61.4%	75.8%	0.372	70
TyG	0.760	0.695~0.816	89.1%	52.4%	0.415	8.78
Gender+Age+TyG	0.857	0.801~0.901	83.1%	79.8%	0.629	–

TyG, Triglyceride Glucose Index.

## 4 Discussion

The main findings of this study highlight the significant association between TyG and the risk of osteoporosis in patients with T2DM.Our results demonstrate that TyG,along with gender and age,serves as an independent predictor of osteoporosis in this population.Specifically,a higher TyG value is associated with an increased likelihood of developing osteoporosis,suggesting that insulin resistance,as measured by TyG,may play a crucial role in the pathogenesis of osteoporosis among individuals with T2DM.Additionally,the predictive model incorporating gender,age,and TyG achieved a high area under the curve(AUC)of 0.857,with a diagnostic sensitivity of 83.1%and specificity of 79.8%.These findings underscore the potential utility of TyG as a simple,cost-effective biomarker for early screening of osteoporosis in clinical settings,particularly among high-risk populations such as elderly patients with long-standing diabetes.

The development of osteoporosis in individuals with T2DM is a complex process primarily associated with several factors, including obesity (20), hyperinsulinemia (21), hyperglycemia (22), advanced glycation end products (23), the metabolic syndrome, the changes of gastrointestinal hormones (24) and renal impairment (25). Furthermore, patients with diabetes often experience hormonal deficiencies, as well as microvascular and neuropathic complications, which further compromise bone nutrition and increase the risk of osteoporosis (26–28). Both T2DM and osteoporosis represent significant global health challenges (29, 30). However, osteoporosis screening remains poorly implemented, resulting in missed opportunities for timely diagnosis and treatment, which can lead to osteoporotic fractures and a substantial decline in quality of life. Patients with concurrent diabetes and osteoporosis generally exhibit a worse prognosis compared to those with diabetes alone, with delayed diagnosis significantly increasing the risk of fractures (31).In our study involving 207 patients with T2DM, 83 individuals were identified as having developed osteoporosis, yielding a prevalence rate of 40%. This finding is consistent with prior research and underscores the urgent need for prompt prevention and treatment strategies for osteoporosis (32).

This study utilized univariate analysis to compare clinical data between the osteoporosis group and the non-osteoporosis group among patients diagnosed with T2DM. The analysis revealed statistically significant differences in gender, age, hemoglobin levels, red blood cells count, total cholesterol levels, and TyG levels between the two groups. Further analysis using binary logistic regression indicated that TyG, gender, and age are independent predictors of osteoporosis in patients with T2DM. While the individual predictive accuracies of gender, age and TyG index are suboptimal (AUC<0.8), the combined model incorporating age, gender and TyG achieved high accuracy with an AUC of 0.857 (95% confidence interval: 0.801-0.901). This combined model significantly outperformed the individual predictors with a diagnostic sensitivity of 83.1% and a specificity of 79.8%.These results suggest that the combined model may serve as a more reliable tool for predicting osteoporosis in patients with type 2 diabetes than using age or TyG index alone.

While the precise mechanism linking osteoporosis in patients with T2DM to TyG remains unclear, it is hypothesized that TyG, as a marker of insulin resistance (IR), plays a significant role (33, 34). The hyperinsulinemic-euglycemic clamp (HEGC) technique is currently regarded as the gold standard for identifying IR. However, due to its complexity and high cost (33), this method is impractical for routine clinical application. The Homeostatic Model Assessment of Insulin Resistance (HOMA-IR) is frequently utilized as an alternative (35), but unlike HOMA-IR, which requires fasting insulin levels and may be less reliable in patients with β-cell dysfunction or on exogenous insulin therapy, the TyG index does not require measurement of insulin, and is therefore more readily available and cost-effective.

In comparison to these methods, TyG provides a simpler and more cost-effective approach for evaluating insulin resistance (IR). Recent research has indicated a significant negative correlation between TyG and bone density, suggesting that TyG plays a pivotal role in assessing IR in relation to bone health (36–38). Furthermore, numerous studies have demonstrated that the accuracy of TyG in evaluating insulin resistance is comparable to that of the hyperinsulinemic-euglycemic clamp (HEGC) and the Homeostatic Model Assessment of Insulin Resistance (HOMA-IR) (39–41). This body of research has identified TyG as a valuable biochemical marker, with receiver operating characteristic (ROC) analysis determining the optimal TyG cutoff value for predicting osteoporosis to be 8.78. This finding indicates that individuals diagnosed with T2DM whose TyG value exceeds 8.78 are significantly more likely to develop osteoporosis.Meanwhile, the predictive model constructed using gender, age, and the TyG index achieved an area under the curve (AUC) of 0.857 (95% confidence interval: 0.801~0.901), with a maximum Youden index of 0.629. The corresponding diagnostic sensitivity was 83.1% and the specificity was 79.8%. This predictive model demonstrates high predictive value in determining whether patients with T2DM have osteoporosis.

Recent studies have further highlighted the broad applicability of TyG as a biomarker for a variety of non-communicable diseases. For example, TyG has been shown to be an important predictor of cardiovascular prognosis, including heart failure, stroke and acute coronary syndromes (42–44). It has also been associated with an increased risk of atrial fibrillation, arterial stiffness, peripheral arterial disease and obstructive sleep apnoea (45–48). These findings highlight the potential of TyG as a universal marker of metabolic disorders and its relevance in identifying individuals at risk for multiple diseases. Our study demonstrates an association between TyG and osteoporosis in patients with T2DM, further supporting the use of TyG as a simple, cost-effective screening tool in clinical practice.

In comparison to traditional screening methods for osteoporosis, such as dual-energy X-ray absorptiometry (DEXA), TyG serves as a simple and cost-effective biomarker that can provide effective screening tools in resource-limited settings. While traditional methods are accurate, they are often expensive and require specialized equipment, whereas TyG measurements can be easily obtained through routine blood tests. To facilitate the application of TyG screening in clinical practice, particularly for high-risk patients such as those with long-term diabetes and elderly individuals, it is recommended that glucose and triglyceride tests be conducted during patient visits. Based on the interpretation criteria for the TyG index, clinicians can ascertain the necessity for further bone density testing. This approach aims to enhance the early identification rate of osteoporosis, ultimately improving patient outcomes. In summary, our study demonstrates that TyG is an independent predictor of osteoporosis in patients with T2DM and can serve as a simple, cost-effective biomarker for early screening in clinical settings.

This study has several limitations: as a single-centre, retrospective, case-control study with a limited sample size, its generalisability may be limited. The analysis relied solely on available medical records, and the presence of missing preoperative data for some patients may introduce bias, potentially affecting the performance of the prediction model. In addition, the lack of external validation of the prediction model limits its applicability in different clinical settings. Future work will include the design of a multi-centre, large-sample prospective study to further validate these findings and improve the accuracy of the prediction model, with the potential to improve the accuracy of outcome prediction by incorporating pathological and radiomic data.

In summary, as we continue to investigate the potential applications of TyG in osteoporosis screening, we anticipate that further advancements in this field will provide new perspectives and solutions for improving the overall health status of individuals with T2DM. Through multidisciplinary collaboration and research, we hope to achieve more effective prevention and treatment strategies in the future, significantly reducing the risk of complications associated with osteoporosis.

## 5 Conclusion

The findings of this study indicate that a higher TyG is associated with an increased risk of osteoporosis in individuals diagnosed with T2DM. Due to its affordability, widespread availability, and ease of implementation, TyG has the potential to serve as a prominent biomarker for the diagnosis of osteoporosis among type 2 diabetic patients in various clinical settings.

## Data Availability

The raw data supporting the conclusions of this article will be made available by the authors, without undue reservation.
